# Convolutional neural network application for supply–demand matching in Zhuang ethnic clothing image classification

**DOI:** 10.1038/s41598-024-64082-9

**Published:** 2024-06-10

**Authors:** Jingyi Ji, Yonghua Lao, Lei Huo

**Affiliations:** 1 School of Art, South China University of Technology, Guangzhou, 510641 People’s Republic of China; 2 National Engineering Research Center for Tissue Restoration and Reconstruction, School of Materials Science and Engineering, South China University of Technology, Guangzhou, 510641 People’s Republic of China

**Keywords:** Supply–demand matching, Convolutional neural network, Zhuang ethnic clothing, Image classification, Visual style, Computer science, Information technology, Software, Statistics

## Abstract

This study aims to design a classification technique suitable for Zhuang ethnic clothing images by integrating the concept of supply–demand matching and convolutional neural networks. Firstly, addressing the complex structure and unique visual style of Zhuang ethnic clothing, this study proposes an image resolution model based on supply–demand matching and convolutional networks. By integrating visual style and label constraints, this model accurately extracts local features. Secondly, the model’s effectiveness and resolution performance are analyzed through various performance metrics in experiments. The results indicate a significant improvement in detection accuracy at different annotation points. The model outperforms other comparative methods in pixel accuracy (90.5%), average precision (83.7%), average recall (80.1%), and average F_1_ score (81.2%). Next, this study introduces a clothing image classification algorithm based on key points and channel attention. Through key point detection and channel attention mechanisms, image features are optimized, enabling accurate classification and attribute prediction of Zhuang ethnic clothing. Experimental results demonstrate a notable enhancement in category classification and attribute prediction, with classification accuracy and recall exceeding 90% in top-k tasks, showcasing outstanding performance. In conclusion, this study provides innovative approaches and effective solutions for deep learning classification of Zhuang ethnic clothing images.

## Introduction

Ethnic clothing serves as a crucial carrier for cultural heritage and identity, playing an irreplaceable role in preserving and inheriting ethnic cultures^1,2^. Zhuang ethnic clothing, as an Important Carrier of Cultural Heritage and Identity, bears rich historical, cultural, and ethnic characteristics. Its features include intricate patterns, vibrant colors, and diverse styles. Zhuang ethnic clothing often employs unique embroidery techniques, incorporating traditional totems, natural elements, and geometric shapes, showcasing a distinctive ethnic style. Additionally, Zhuang ethnic clothing boasts a rich array of colors, often dominated by bright hues such as red, green, and yellow, reflecting the people’s love for life and their positive spirit. Moreover, the styles of Zhuang ethnic clothing are highly diverse, encompassing both men’s robes and jackets and women’s long skirts and short blouses, with each style reflecting different social statuses, ages, and occasions. However, it is precisely this complex diversity that poses challenges for the segmentation and classification of Zhuang ethnic clothing images. Firstly, the patterns in Zhuang ethnic attire are often intricate, with overlapping and intertwining elements, making image segmentation difficult. Secondly, the rich colors of Zhuang ethnic clothing present complex color distributions in images, requiring algorithms to accurately identify and extract regions of different colors. Furthermore, the diverse styles also increase the difficulty of image classification, necessitating algorithms capable of recognizing and distinguishing between different styles of clothing^3^. Therefore, addressing the pressing issue of how to utilize advanced Convolutional Neural Network (CNN) technology to enhance the accuracy of Zhuang ethnic clothing image classification becomes imperative.

In the field of image classification, CNN technology has achieved remarkable success. However, research on clothing image classification within specific cultural contexts remains relatively scarce. Current studies predominantly focus on general image classification tasks, overlooking the cultural differences and uniqueness of ethnic clothing images^4,5^. Additionally, most algorithms exhibit certain shortcomings when considering supply–demand matching, failing to exploit the unique features of ethnic clothing images and consequently limiting the improvement of classification performance. Therefore, it is necessary to conduct in-depth research on the features of Zhuang ethnic clothing images, optimize convolutional network models, and adapt them to image classification tasks under different supply–demand conditions. This study aims to fill the gap in existing research by introducing CNN applications that consider supply–demand alignment. It focuses on how to integrate the cultural characteristics of Zhuang ethnic clothing images, optimize CNN models, and improve the accuracy and robustness of image classification. This study emphasizes targeted design to address the complexity and diversity of Zhuang ethnic attire. By overcoming the limitations of existing research, this experiment seeks innovative breakthroughs in the classification of Zhuang ethnic clothing images, providing strong support for the protection and inheritance of ethnic clothing culture^6^.

## Literature review

As society increasingly values ethnic culture, the preservation and inheritance of traditional culture have become increasingly important. Against this backdrop, ethnic clothing, as one of the cultural heritages, has garnered significant attention, with its historical, cultural, and ethnic characteristics becoming focal points of interest. Simultaneously, with the acceleration of globalization, people have begun to realize the vital role that the ethnic clothing industry plays in environmental protection and promoting sustainable development. By integrating the concepts of low-carbon, green, and digitalization, exploring the development path of the ethnic clothing industry not only contributes to the inheritance and promotion of ethnic culture but also fosters green production and digital innovation, paving the way for the sustainable development of the ethnic clothing industry^7–9^. Zhang et al. (2021) ^10^ introduced a deep learning-based image classification method for ethnic clothing using CNN. By incorporating multiscale convolution and pooling operations into the network structure, they comprehensively captured features of clothing images, effectively improving classification accuracy. Zhou et al. (2022) ^11^ embraced a multimodal fusion approach, designing a clothing image classification model that integrated both image and text information. By simultaneously considering images and relevant text descriptions, the model better understood the context and cultural features of clothing images, achieving more accurate classification results. Nocentini et al. (2022) ^12^ proposed an ethnic clothing image classification method based on transfer learning. By transferring knowledge from a model trained in the source domain to the target domain, this approach effectively mitigated the issue of scarce data, enhancing performance in clothing image classification. Jia & Liu (2021) ^13^, leveraging the concept of generative adversarial networks, introduced an adversarial training-based model for ethnic clothing image classification. Through adversarial training, the model learned more discriminative feature representations, enhancing the distinguishability of similar and dissimilar clothing thereby improving classification accuracy. Wang (2023) ^14^ introduced the concept of iterative clustering, designing an automated optimization algorithm for ethnic clothing image classification. This algorithm adaptively adjusted feature representations through multiple iterations of clustering processes, progressively optimizing the classification model and improving performance across different Zhuang ethnic clothing types. Jain et al. (2021) ^15^ presented a clothing image classification method based on Graph Convolutional Network (GCN). By modeling relationships between clothing images, GCN better captured local and global information, improving the robustness and accuracy of classification. Zhou et al. (2022) ^16^, applying domain adaptation techniques from transfer learning, proposed an adaptive clothing image classification model. This model adaptively adjusted feature representations under different supply–demand conditions, enhancing generalization performance in practical applications. Sulthana et al. (2020) ^17^ introduced image generation models into clothing image classification, presenting a generative model-based active learning approach. This method effectively enhanced the model’s capability to classify edge cases and complex samples by actively generating challenging samples, strengthening overall robustness. Juxiang Zhou & Gan (2018) ^18^, focusing on the characteristics of ethnic clothing images, optimized CNN using a feature aggregation weighting method. They further improved the performance of ethnic clothing image retrieval by employing a reordering strategy based on diffusion processes, providing new insights and technical references for researchers in the field of ethnic clothing image retrieval. Juan (2021) ^19^ conducted texture segmentation and automatic matching under a two-layer model based on histogram distribution, enhancing and optimizing the texture information of traditional ethnic clothing patterns. By extracting edge contour feature points of traditional ethnic clothing patterns, they achieved recognition of embroidery features in traditional ethnic clothing. Zhang & Shen (2022) ^20^ proposed a weighted graph tree representation method for the nonlinear deviation transfer process of ethnic clothing. They analyzed business links and information related to modeling ethnic clothing deviation transfer, establishing hierarchical relationship trees, methods for expressing ethnic clothing orders, and deviation expression methods, making certain contributions to the study of ethnic clothing. Lei et al. (2020)^21^ transferred the deep convolutional network Visual Geometry Group (VGG) model to ethnic clothing recognition tasks, integrating local feature extraction to achieve ethnic clothing image recognition. The study showed that in specific garment recognition tasks, utilizing local feature extraction techniques can help algorithms better understand and characterize the unique features of garments, thereby improving recognition accuracy and robustness.

In summary, significant progress has been made in recent years in the field of ethnic clothing image classification, covering a variety of methods and technologies. Deep learning techniques such as CNN have been widely applied in ethnic clothing image classification, effectively improving classification accuracy through methods such as multi-scale convolution and pooling operations. Additionally, some studies have further enhanced classification performance through techniques such as multimodal fusion, transfer learning, and generative adversarial networks, while also addressing issues such as data scarcity and generalization performance. Furthermore, specific optimization methods have been proposed for the characteristics of ethnic clothing images, such as complexity and diversity, including feature aggregation weighting, iterative clustering, and graph convolutional networks, to further improve classification performance. These studies not only provide new insights and technical references for the field of ethnic clothing image retrieval but also make important contributions to the preservation and inheritance of ethnic clothing culture. However, previous research has mainly focused on ethnic clothing image classification, leaving a relatively limited study on Zhuang ethnic clothing images.

### Design of a CNN-based algorithm for Zhuang ethnic clothing image classification

#### Zhuang ethnic clothing

**Figure 1:**
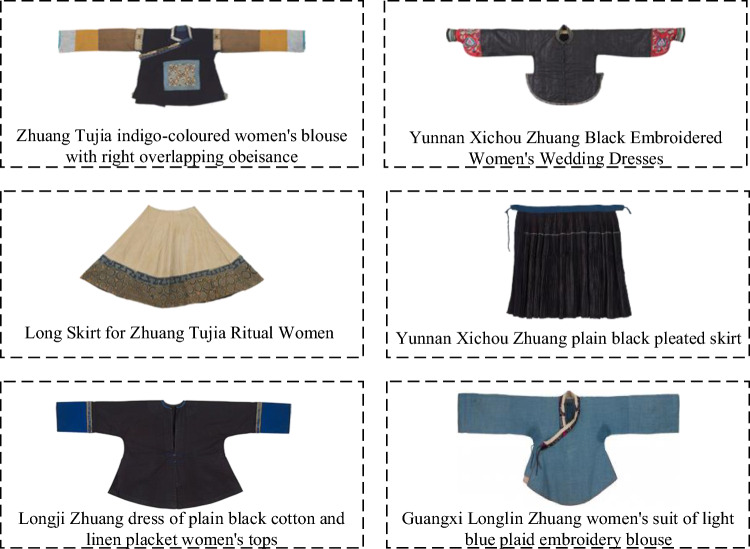
Zhuang ethnic clothing.

Traditional attire of the Zhuang people predominantly features three main colors: blue, black, and brown. Renowned for its unique style and rich cultural characteristics, Zhuang clothing is famous worldwide. Men often wear black or blue buttoned short shirts, paired with loose Chinese-style trousers and traditional grass shoes or cloth shoes with cut-out designs, topped with a black cloth cap. Women typically wear black headscarves, diagonally buttoned tops, and wide black trousers, complemented with exquisite embroidered shoes and Zhuang brocade cylindrical bags. They often adorn themselves with silver necklaces, silver bracelets, and other accessories, showcasing a strong ethnic flavor. The commonly seen Zhuang brocade in their attire, known for its exquisite craftsmanship and unique patterns, adds a distinctive charm to the entire ensemble, becoming a highlight of Zhuang culture. Typical traditional Zhuang attire is illustrated in Fig. 1.

### Resolution of Zhuang ethnic clothing images based on supply-demand matching and convolutional networks

Zhuang ethnic clothing, characterized not only by its structural complexity but also by a distinctive visual style, presents significant differences in color blocks and style categories within images. The intricate local features, coupled with numerous semantic labels, add complexity to the resolution task. Such intricacies result in a noticeable “semantic gap” between low-level features and high-level attributes, impacting the accuracy and precision of current resolution methods^22,23^.

The semantic labels in Zhuang ethnic clothing images refer to the annotation of semantic information in different regions of the clothing images, describing the composition and characteristics of the attire. These labels include tags for upper body, lower body, overall, accessories, and other regions, with each label corresponding to possible clothing components or accessories in that area. For instance, the upper body region may include items such as tops, vests, or short shirts, while the accessories region may include items like scarves, belts, or headscarves. Label pairs indicate the potential relationships or combinations between different semantic labels. In Zhuang ethnic clothing images, different clothing components and accessories are often interrelated. For example, a headscarf may be associated with hair or headwear, while a belt may be linked to trousers or skirts. Semantic annotation is the process of describing and explaining the clothing components, accessories, and other related items appearing in an image. Through semantic annotation, the content and structure of the image can be understood more clearly, facilitating further image interpretation and analysis. Key points refer to the positions in the image that hold important semantic or feature information, typically used to describe and locate key parts or regions in the image. In Zhuang ethnic clothing images, key points may include important areas such as the neckline, cuffs, waist, as well as the locations of accessories. By detecting and marking key points, the local features and structural information in the image can be more accurately understood, aiding in subsequent classification, recognition, and analysis tasks.

**Figure 2:**
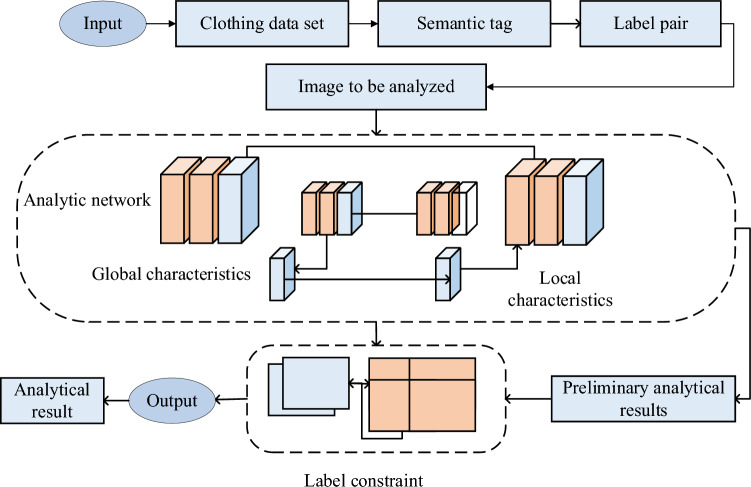
Resolution process of Zhuang ethnic clothing images based on visual style and label constraints.

To address this issue, this section integrates the concept of supply-demand alignment into the parsing model to better adapt to the cultural characteristics and structural information of Zhuang ethnic clothing. Experiments involve the targeted design of parsing models considering the cultural demands of different regions to tackle the challenges posed by the diversity of Zhuang clothing. Consequently, a novel Zhuang ethnic clothing image parsing model is proposed in this study. The model is based on visual style and label constraints. This ethnic clothing image parsing model aims to achieve in-depth parsing and understanding of Zhuang ethnic clothing images by comprehensively considering the visual style and relevant label information of the image. By integrating visual style, texture, color, and other visual features in the image can be captured, while label constraints provide prior knowledge about clothing categories and attributes. This integrated parsing model effectively interprets the semantic meaning of the image and extracts important features. Through this model, Zhuang ethnic clothing images can be analyzed and recognized more accurately, providing a powerful tool and method for the preservation, inheritance, and research of ethnic clothing. Please refer to Fig. 2 for a detailed illustration of the specific construction process.

Figure 2 illustrates the image resolution process, which can be divided into two key steps: firstly, utilizing training images with semantic labels and annotations to establish a visual style branch network, thereby integrating the learning of local features of Zhuang ethnic clothing; secondly, utilizing the label constraint network to enhance the resolution results. The specific contents of each module in the process are detailed below.

#### Visual style network

(1) Seg Net

In this study, Seg Net is employed as the foundational resolution model, which is a type of deep full CNN. Its overall framework comprises an encoder network, a corresponding decoder network, and a pixel-level classification layer. The structure of the encoder network is similar to the VGG-16 network. The structure of all encoders includes convolutional layers, batch normalization layers, and ReLU functions, with a window size of 20 and a stride of 2 for max pooling. This network framework is relatively straightforward, with a one-to-one correspondence between the encoder and decoder^24–26^.

(2) Side Branch Network

**Figure 3:**
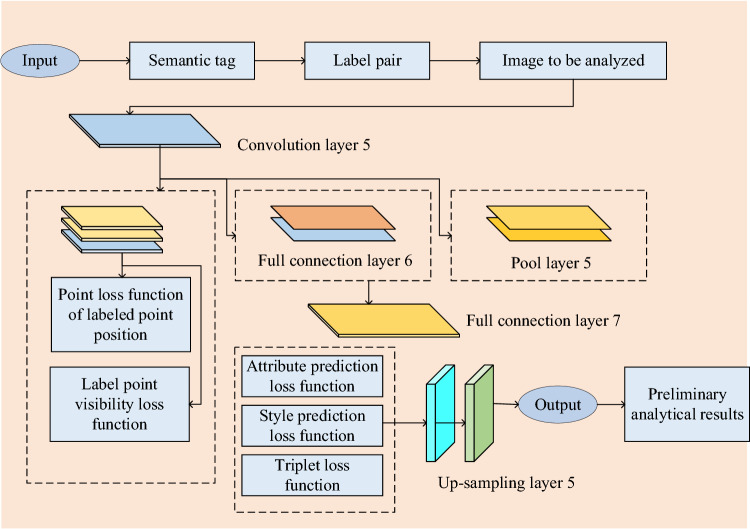
Visual style side branch network.

The side branch network constructed and utilized in this study is depicted in Fig. 3.

In the visual style network section of this study, local and global features are extracted through channel-wise connections. This connection is achieved by feeding the outputs of two fully connected layers as position and visibility information into the side branch network. Firstly, these pieces of information are utilized to extract clothing annotation points, acquiring the local features of the attire. Simultaneously, using the encoder-decoder network structure, the entire image undergoes pixel-level classification to obtain the global features of the clothing. Subsequently, the extracted local features and global features are concatenated at the channel level to obtain a comprehensive visual style feature matrix. Based on this, attributes and styles are classified, and triplet loss is utilized to assist in learning the image’s style features. Finally, predicted style features are returned to the Seg Net network through deconvolutional layers, enabling the network to comprehensively consider both local and global features, thereby achieving more accurate image style classification.

First, the positioning calculation of key points is computed using L_2_ regression loss as shown in Eq. (1):

1 $${L}_{location}=\sum_{i=1}^{|T|}{||{v}_{i}\cdot (\widehat{{l}_{i}}-{l}_{i})||}_{2}^{2}$$

In Eq. (1), *T* refers to the scale of the images involved in training, $\widehat{{l}_{i}}$ denotes the correct position information of the *i*th image that has been labeled correctly, ${l}_{i}$ represents the predicted value of the position of the labeled point, and ${v}_{i}$ represents the visibility vector of the labeled points in the *i*th training image, where 1 denotes visible and 0 denotes invisible.

Next, based on the constructed semantic label content for Zhuang ethnic clothing, the loss of attribute prediction is calculated using cross-entropy loss function:

2 $$L=\sum_{i=1}^{|T|}({\gamma }_{1}\cdot{a}_{i}logp\left({a}_{i}|{g}_{i}\right)+{\gamma }_{2}\cdot(1-{a}_{i})\text{log}(1-p\left({a}_{i}|{g}_{i}\right)))$$

In Eq. (2), ${\gamma }_{1}$ and ${\gamma }_{2}$ refer to two different coefficients; ${g}_{i}$ denotes the *i*th image, and ${a}_{i}$ represents the optimized semantic label vector information corresponding to the *i*th image.

Due to the existence of four annotated points, the number of annotated points varies in different images. Additionally, because the dataset contains half-body images and some annotated points may be occluded, resulting in differences in the visibility of each annotated point. Therefore, by using softmax loss to define the loss function for the visibility of annotations, its calculation is as shown in Eq. (3):

3 $${L}_{v}=-\sum_{j=1}^{K}{y}_{j}log{P}_{vj}$$

In Eq. (3), ${y}_{j}$ denotes a 1*K vector, where *K* is the number of annotation points. Meanwhile, ${P}_{vj}$ represents the visibility probability of the *j*th annotated point.

Following this, the loss situation of style prediction is calculated using the softmax loss function for multi-label classification, as shown in Eq. (4):

4 $${L}_{style}=-\sum_{k=1}^{7}{w}_{j}log{s}_{i}$$

Finally, by employing the triplet loss function to enhance the distance constraint between positive and negative sample training images in clothing images, this study accomplishes feature learning and annotation of clothing local regions.

5 $${L}_{pair}=\sum_{i=1}^{|T|}\text{max}\{0,m+d\left({g}_{i},{g}_{i}^{+}\right)-d({g}_{i},{g}_{i}^{-})\}$$

In Eq. (5), *d* represents the distance function, ${g}_{i}$ denotes the *i*-th training image, ${g}_{i}^{+}$ denotes an image similar to ${g}_{i}$, ${g}_{i}^{-}$ denotes an image dissimilar to ${g}_{i}$, and m is the margin coefficient.

**Figure 4:**
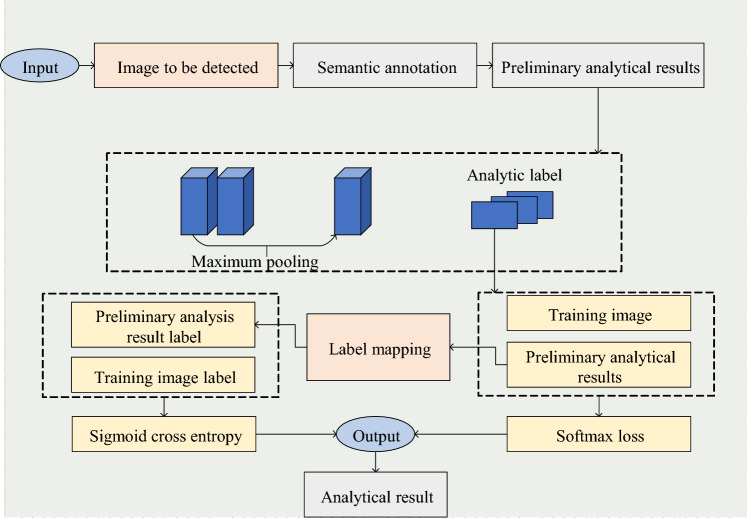
Flowchart of label constraint.

*Label constraint* Due to the differences between pixel matching and label matching, this dissimilarity can lead to confusion in label annotation. Given this challenge, this study drew inspiration from the conditional progressive network. It utilized the method of establishing label vectors to minimize label redundancy, simplify the complex relationships between labels, and mitigate parsing errors resulting from label confusion. Figure 4 provides a detailed illustration of the label constraint process.

Figure 4 presents the processing flow of label constraints. Firstly, using a label mapping function, the initially parsed image is transformed into a label vector where each element indicates whether the parsing result includes the corresponding label. Subsequently, the label vector is adjusted by comparing it with the actual semantic labels of the training set, effectively avoiding the probability values of redundant labels. Finally, the image-level labels are added as a constraint to the loss function to optimize the parsing result^27^.

#### Experimental design

(1) Data collection

The images selected for this study are sourced from publicly available museums and cultural exhibitions, primarily collected from the National Costume Museum of Beijing Institute of Fashion Technology, totaling 500 samples. These samples mainly consist of female attire because women’s clothing exhibits a rich variety, and their accompanying accessories possess strong ethnic characteristics, making them representative examples. Additionally, a small portion of male clothing samples is included to comprehensively showcase the diversity and richness of Zhuang ethnic clothing. In this study, 70% of the dataset is used for training the model to ensure that it adequately learns the features and patterns present in the dataset. Furthermore, 15% of the original images in the dataset are used for model validation to monitor the model’s performance during training and conduct optimization operations such as hyperparameter tuning. Finally, the remaining 15% of the data is utilized for testing the model’s performance to assess its generalization ability on unseen data. All images are uniformly resized to a resolution of 360 × 480 pixels, and their format is standardized as JPG to ensure data consistency and ease of processing.

(2) Semantic Label Design

Initially, based on the structural styles of Zhuang ethnic clothing in China, the experiment meticulously divided different parts of the human body, establishing a new and comprehensive semantic label system for Zhuang ethnic clothing. Specifically, there are 7 labels for the upper body, 5 for the lower body, 2 global labels, 11 labels for accessories, and 3 for other aspects. This detailed classification system is listed in Table 1.

**Table 1:** Semantic labels for Zhuang ethnic clothing.

Body part	Semantic Labels	Number
Upper Body	Top, Waistcoat, Short Shirt, Shawl, Vest, Shirt, Suit Skirt	7
Lower Body	Pants, Long Skirt, Shorts, Shoes, Boots	5
Global	Long Robe, Dress	2
Accessories	Tube Scarf, Belt, Bag, Headscarf, Waist Knife, Hat, Headband, Collar, Apron, Headdress, Handkerchief	11
Other	Background, Skin, Hair	3

### Classification of Zhuang ethnic clothing images based on key points and attention mechanisms

The previous section presents the parsing algorithm for Zhuang ethnic clothing images, explaining that in the feature extraction stage, CNN suitable for clothing images are utilized, including network layer structures adjusted for the characteristics of Zhuang clothing. Specific key point detection networks are designed for extracting local features of Zhuang clothing images to accurately locate key areas such as patterns and motifs, thus better extracting local features. In this section, a combination of key point extraction and channel attention mechanism is employed for classifying parsed images. The channel attention mechanism is introduced to ensure that the network focuses on the feature channels that have the most significant impact on classification results, thereby improving classification accuracy. At the end of the network, a multi-branch structure is adopted to handle different tasks separately, such as category classification and attribute prediction. This design makes the network more flexible, capable of simultaneously meeting the requirements for accurate identification of image categories and attributes. Through these customized designs, the network can better adapt to the characteristics of Zhuang clothing images, enhancing the performance and effectiveness of the network in Zhuang clothing classification tasks.

**Figure 5:**
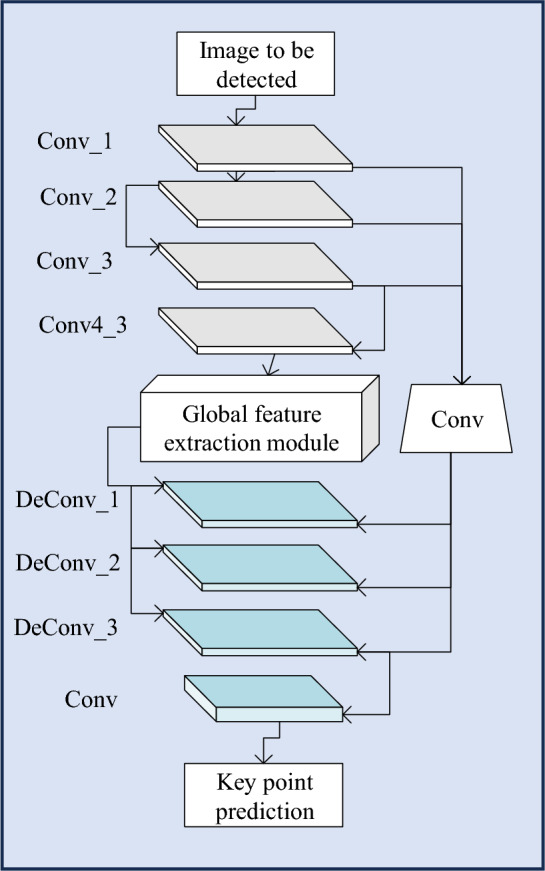
Structure of the key point detection network.

*Image key point detection algorithm.* The input of the key point detection network is derived from the Conv4_3 layer feature map of the baseline network VGG16. A concatenated network is employed to transform the low-resolution feature map into a high-resolution feature map using deconvolutional techniques. In contrast to traditional upsampling methods, this study utilizes deconvolution operations with learnable parameters. These parameters enable the network to restore the feature map in a learned manner. In order to predict the positions of clothing key points more effectively, a multiscale fusion strategy is adopted, combining shallow high-resolution feature maps with deep upsampled feature maps^28^. Additionally, the experiment designs a Key Point Attention Network to achieve key point detection. This network consists of three main modules: the global feature extraction module, the deconvolution module, and the multiscale fusion module. The overall network structure is illustrated in Fig. 5.

#### Image classification based on key points and channel attention

(1) Channel attention

In neural networks, each layer’s feature map contains multiple channels, and these channels contribute differently to the classification results. To handle this diversity more finely, a channel attention mechanism is introduced to assign weights to different channels in the feature map. This ensures that channels with a more significant impact on the classification results receive higher weights^29^. In comparison, those with a lesser impact receive lower weights. For a feature map of size *H *× *W *× *C*, a one-dimensional weight vector of size 1 × 1 × *C* can be used in the channel attention mechanism. Each element in this vector represents the weight of the corresponding channel in the original feature map, and a new feature map is obtained by element-wise multiplication.

**Figure 6:**
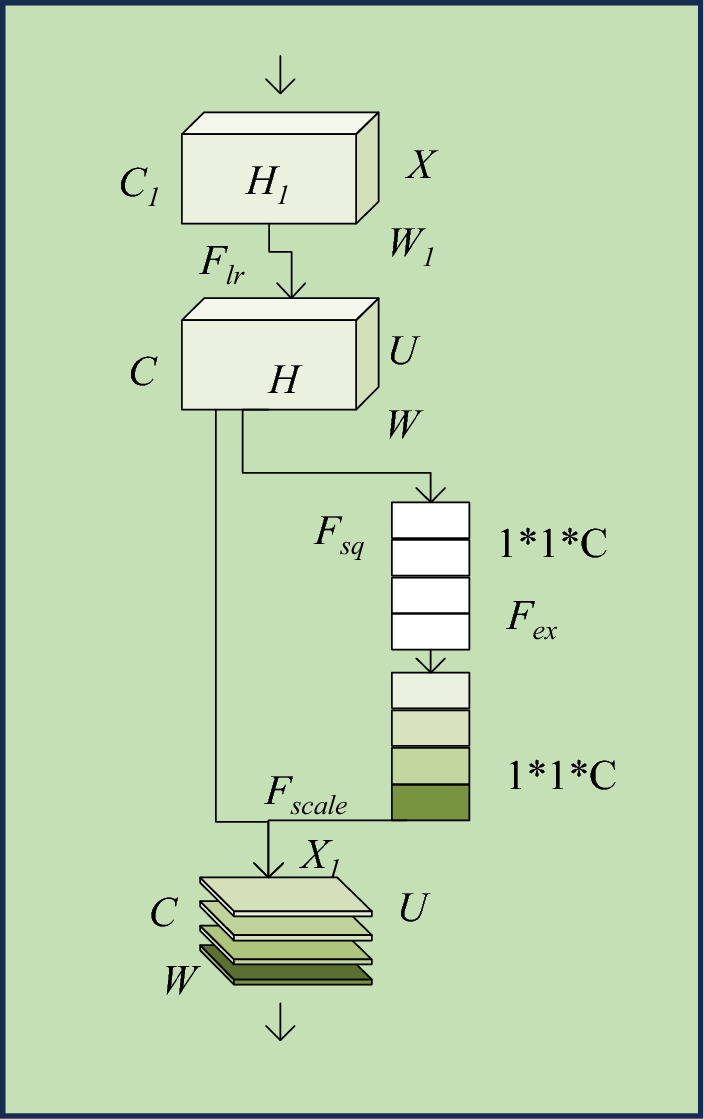
Structure of the SE block.

The channel attention module selected in this study is the SE Block, whose structure is depicted in Fig. 6.^30^

Figure 6 illustrates the structure of the SE Block. The SE Block structure mainly consists of two core components, denoted as *Fsq* and *Fex*. *Fsq* represents the Squeeze operation, which compresses each two-dimensional feature map into a single value. On the other hand, *Fex* represents the Excitation operation, which calculates the weight values for each channel, thereby determining the importance of different channels.

(2) Clothing image classification algorithm based on key points and channel attention

**Figure 7:**
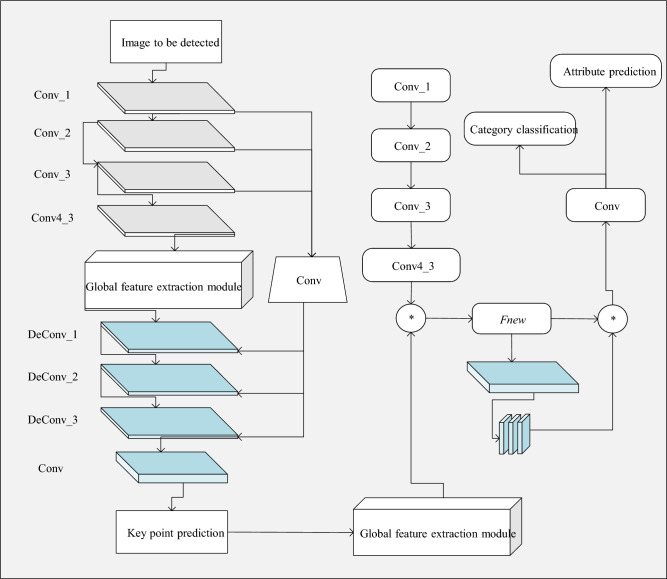
Structure of the image classification algorithm.

The structure of the clothing image classification algorithm based on key points and channel attention, designed in this study, is depicted in Fig. 7.

Figure 7 illustrates the structure of the image classification algorithm. This algorithm is based on key point localization and incorporates key point attention, introducing it into the main network to enhance the quality and accuracy of the feature map. The purpose of this crucial step is to highlight the importance of key point regions in the feature map, aiming to obtain more refined feature maps. In addition to focusing on spatial positions, since each image in a set of feature maps contributes differently to the final classification task, this study also introduces a channel attention module. Through a series of calculations, this module analyzes the input feature maps, determining the weight of each feature map and indicating its level of importance throughout the entire network. The experiment obtains optimized feature maps through the learning and filtering processes of these two attention modules. In the final stage of the network, this study employs two independent branches for category classification and attribute prediction.

#### Experimental design

(1) Experimental environment and dataset

The dataset used in this study is consistent with Section 3.1, and the configuration of the experimental environment is detailed in Table 2.

**Table 2:** Experimental environment.

Environment Type	Parameter	Configuration
Hardware environment	Host	4 × RTX 3090
	Host	AMD Ryzen 9 5900X
	Memory	128G
	Storage	1 TB NVMe Solid State Drive
Software environment	Operating System	Windows 11
	Programming Language	Java
	Deep Learning Framework	TensorFlow2.5

(2) Evaluation criteria

For category classification, the experiment employed the top-*k* classification accuracy as the evaluation metric. Here, top-k refers to selecting the most likely *K* results in the predicted outcomes, *i*.*e*., the categories with probability rankings in the top *k*. If these *k* categories include the true label, it is considered a correct prediction; otherwise, it is deemed incorrect. The top-*k* classification accuracy calculates the proportion of correctly predicted images in the top-*k* among all test images. This experiment conducted classification accuracy tests for both top-3 and top-5 scenarios.

Regarding attribute prediction, the experiment utilized top-*k* recall as the evaluation criterion. This study counted the number of matching attributes within the top-*k* by ranking attribute scores. Taking *k* as 5 for example, if only 1 attribute among the top 5 matches the actual attributes of the clothing, and the garment has a total of 2 attributes, then the recall rate is 50%. The final presentation of experimental results involves averaging the recall rates across multiple samples. Similarly, the experiment examined the recall rates for top-3 and top-5.

## Experimental results and analysis

### Effectiveness of the convolutional network-based zhuang ethnic clothing image parsing model

(1) Effectiveness analysis

**Figure 8:**
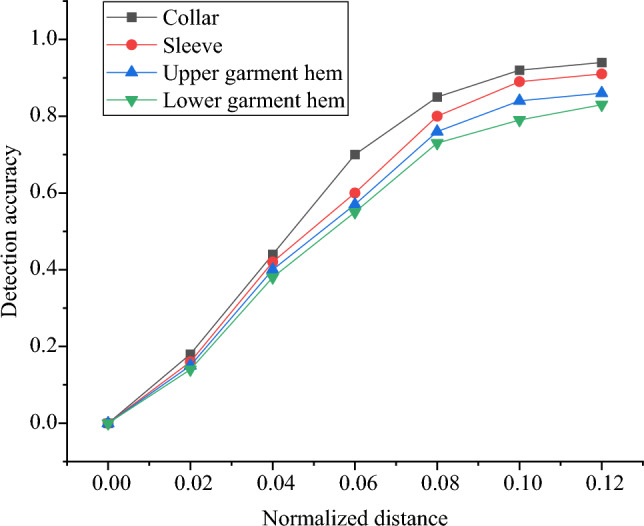
Detection accuracy of different annotated pairs.

In the visual style network, it is crucial to locate the position information of the annotated pairs to capture and learn the features of various parts of ethnic clothing. The experimental results indicate variations in the detection accuracy of annotated points under different thresholds. Specific details are presented in Fig. 8.

Figure 8 illustrates the detection accuracy of annotated points under different thresholds.

Figure 8 illustrates that when the normalized distance reaches 0.1, the detection accuracy of all four annotated points is generally around 0.8 or even higher. However, as the threshold continues to increase, the detection accuracy shows a stabilizing trend. Moreover, the detection rate for the collar is significantly higher than that for the sleeves, upper garment hem, and lower garment hem. This is mainly because the collar is located at the neck, experiencing less occlusion and deformation. Additionally, other annotated points are more susceptible to the influence of body joints, leading to deformation and occlusion.

(2) Performance analysis and method comparison

**Figure 9:**
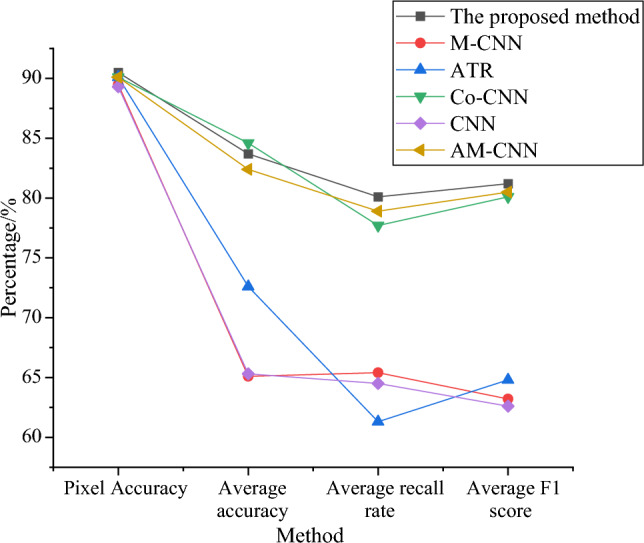
Comparison of detection accuracy under different methods.

This study evaluates the performance of minority ethnic clothing parsing using pixel accuracy, average precision, average recall, and average F_1_ score. Table 3 compares the performance of our approach with Multiscale Convolutional Neural Networks (M-CNN) ^31^, ATR (Attention to Regions) ^32^, and Collaborative Convolutional Networks (Co-CNN) ^33^, and compares it with the single module used in this study, analyzing the performance of different methods on the dataset used in this study. The comparison results are shown in Fig. 9.

**Table 3:** Comparative results of clothing category classification and attribute prediction.

Method	Classification		Shape	
	top-3	top-5	top-3	top-5
WTBI	43.7	66.3	23.4	31.3
DARN	59.5	79.6	35.9	46.9
Fashion Net	82.6	90.2	39.4	48.6
The proposed method	91.2	95.6	59.4	68.4

In Fig. 9, this method achieves a pixel accuracy of 90.5%, significantly outperforming M-CNN (89.5%), ATR (90.1%), and Co-CNN (90.1%). Regarding average precision, our method achieves 83.7%, surpassing both M-CNN and ATR methods, which achieve 65.1% and 84.6%, respectively. The proposed method also demonstrates high levels of average recall and average F1 score, reaching 80.1% and 81.2%, respectively. The performance of the single CNN method is inferior in all aspects compared to our method. The Attention Mechanism-CNN (AM-CNN) method performs well in pixel accuracy, average precision, average recall, and average F1 score, but its performance is slightly lower than the proposed method. Overall, the proposed method exhibits significant advantages in all performance metrics, demonstrating its powerful capability in minority ethnic clothing parsing. In contrast, there are noticeable performance gaps between M-CNN, ATR, Co-CNN, CNN, AM-CNN, and the proposed method in certain indicators, highlighting the outstanding performance of our method.

### Performance of clothing classification algorithm based on keypoints and channel attention

For category classification and attribute prediction, this study compares the proposed method with WTBI (Weakly-Supervised Transformer-Based Image Retrieval) ^34^, DARN (Dual Attention Recurrent Network) ^35^, and Fashion Net^36^. The experimental results are presented in Table 3.

Table 3 indicates that the proposed network model excels in clothing classification and attribute prediction, achieving significant breakthroughs in accuracy. In terms of category prediction, not only do the top-3 and top-5 accuracies surpass 90%, but the top-5 accuracy even breaks the barrier of 95%. Additionally, for shape feature prediction, the top-3 accuracy reaches 59.4%, while the top-5 accuracy reaches 68.4%, fully aligning with CNN’s outstanding feature extraction capabilities.

### Comparison of recognition performance under different methods and datasets

**Figure 10:**
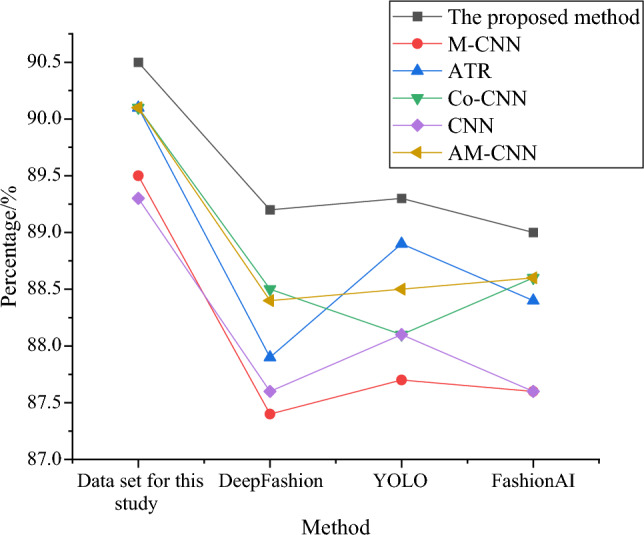
Comparison of detection accuracy on different datasets.

For the recognition performance on different datasets, this study compares with the DeepFashion database, YOLO ethnic clothing recognition dataset, and FashionAI dataset to analyze the universality and versatility of the proposed method. The DeepFashion database, YOLO ethnic clothing recognition dataset, and FashionAI dataset are three important data resources commonly used in fashion and clothing-related research. The DeepFashion database contains a large number of fashion images and annotations, which can be used for tasks such as clothing detection, classification, and retrieval. The YOLO ethnic clothing recognition dataset is designed for ethnic clothing recognition, trained and tested using the YOLO algorithm, providing images and annotations specifically tailored for ethnic clothing. The FashionAI dataset is a comprehensive fashion dataset that includes various information such as clothing attributes, key points, accessories, etc., suitable for applications such as fashion recommendation and clothing style analysis. The experimental results are shown in Fig. 10.

In Fig. 10, there are certain differences in detection accuracy among different methods on various datasets. In the dataset used in this study, the proposed method achieves the highest detection accuracy, reaching 90.5%. Meanwhile, in the DeepFashion, YOLO, and FashionAI datasets, the performance of various methods is roughly similar, with detection accuracy around 89%. The data indicate that the proposed method performs excellently on the dataset used in this study and maintains a high level of performance on other datasets as well.

## Conclusion

This study addresses the complexity and diversity of Zhuang ethnic clothing images by designing and implementing a parsing and classification algorithm based on supply–demand adaptation and convolutional networks. By integrating the concept of supply–demand adaptation into the parsing model, it proposes an innovative Zhuang ethnic clothing image parsing model that combines visual style and label constraints. This model effectively enhances the resolution accuracy of local features and semantic information by synergizing visual style networks and label constraint networks. Experimental results demonstrate that the proposed method significantly outperforms other comparison methods in terms of parsing performance, achieving higher pixel accuracy, average precision, average recall, and average F1 score. In terms of clothing classification algorithms based on key points and channel attention, the proposed model exhibits remarkable improvements, achieving excellent classification and attribute prediction performance. This study contributes to the accurate classification of Zhuang ethnic clothing images, achieving precise classification of the complex structure and unique visual style of Zhuang ethnic clothing. It provides innovative methods and effective solutions for Zhuang ethnic clothing image classification, further improving classification accuracy and attribute prediction capabilities, and offering important references and insights for research and applications in related fields. Through innovative convolutional network structures, this study overcomes the challenges posed by the complexity of Zhuang clothing images. By integrating visual style and label constraints, precise extraction of local features is achieved, and a classification algorithm based on key points and channel attention is proposed to further optimize image features, achieving more accurate classification and attribute prediction. Thus, this study not only applies CNN but also represents an innovative exploration in methodology, bringing new contributions to the field of Zhuang clothing image classification.

However, there are still some limitations, including the relatively limited scale of the dataset and the need to improve the generalization ability for other ethnic clothing. Future studies can focus on expanding the dataset, further optimizing the model structure, and considering the integration of other ethnic characteristics to achieve more comprehensive and accurate clothing image parsing and classification.

## Data Availability

The data presented in this study are available on request from the corresponding author.
